# Aurora kinase A (AURKA) promotes the progression and imatinib resistance of advanced gastrointestinal stromal tumors

**DOI:** 10.1186/s12935-021-02111-7

**Published:** 2021-07-31

**Authors:** Xiaobin Cheng, Jinhai Wang, Sen Lu, Weina Fan, Weilin Wang

**Affiliations:** 1 Department of Colorectal Surgery, The First Affiliated Hospital, Zhejiang University School of Medicine, Hangzhou, People’s Republic of China; 2 Department of Intensive Care Unit, The First Affiliated Hospital, Zhejiang University School of Medicine, Hangzhou, People’s Republic of China; 3 Department of Hepatobiliary and Pancreatic Surgery, The Second Affiliated Hospital, Zhejiang University School of Medicine, #88 Jiefang Road, Hangzhou, Zhejiang People’s Republic of China

**Keywords:** AURKA, Progression, Imatinib resistance, Gastrointestinal stromal tumors

## Abstract

**Background:**

Gastrointestinal stromal tumor (GIST) is a common tumor that originates from the alimentary system mesenchyme. Compared to typical gastrointestinal carcinomas, GISTs exhibit unique malignant behaviors. Bioinformatic tools and subsequent experiments were applied to investigate novel targets involved in GIST progression and imatinib resistance.

**Methods:**

Differences in gene expression profiles between advanced and nonadvanced GISTs were comprehensively analyzed based on the Gene Expression Omnibus (GEO) dataset GSE136755. A protein–protein interaction (PPI) network was constructed to identify the potential target gene. Gene set enrichment analysis (GSEA) was used to elucidate relevant biological events related to the target gene based on the GSE47911 dataset. Subsequently, immunohistochemistry and Kaplan–Meier analysis were performed to validate the prognostic value of the target gene in GISTs. Overexpression of the target gene was conducted to analyze its function in the proliferation, apoptosis, and imatinib resistance of GIST/T1 cells.

**Results:**

In the current study, a total of 606 differentially expressed genes (DEGs) were screened based on the GSE136755 dataset, and the upregulated DEGs in advanced GISTs were mainly involved in cell division through functional annotations. The intersecting hub gene, Aurora kinase A (AURKA), was identified by degree and bottleneck algorithms. GSEA revealed that AURKA was involved in cell cycle-related biological processes. Analysis of the Oncomine and GEPIA databases revealed a pattern of elevated AURKA expression in most human malignances. Clinical assays demonstrated that AURKA could be an independent prognostic factor for GISTs. Additionally, overexpression of AURKA was experimentally demonstrated to promote cell proliferation, inhibit cell apoptosis, and enhance imatinib resistance in GIST/T1 cells.

**Conclusions:**

These findings indicated that overexpression of AURKA promoted GIST progression and enhanced imatinib resistance, implying that AURKA is a potential therapeutic target for GISTs.

**Supplementary Information:**

The online version contains supplementary material available at 10.1186/s12935-021-02111-7.

## Background

Gastrointestinal stromal tumor (GIST) is the most common mesenchymal tumor of the alimentary system and originates from the interstitial cells of Cajal (ICC) [1]. Approximately 71% of GISTs present with KIT (71%) or PDGFRα (14%) mutations, and 10–15% of GISTs do not have KIT or PDGFRα mutations, referred to as KIT/PDGFRα wild type GISTs [2, 3]. The malignant potential of GISTs is stratified based on tumor size, mitotic index, and location according to the modified NIH criteria [4]. Based on these criteria, GISTs are classified as high-risk, intermediate-risk, low-risk, and very low-risk. The 5-year survival rate of patients with advanced GIST is between 35 and 65% [5]. The main therapeutic option for primary localized GISTs is surgical resection. However, the recurrence rate for GISTs, even after complete surgical resection, is 40–80% [5]. The median time to recurrence for most patients is approximately 12–16 months [6]. Treatment with imatinib, a tyrosine kinase inhibitor (TKI) that targets KIT and PDGFRα, has improved the prognosis of GIST patients. However, when used to eliminate mature GIST cells, imatinib has limited efficacy, and studies have revealed that GIST persists with prolonged TKI therapy [1]. Due to acquired resistance to imatinib, approximately 85–90% of patients with GIST experience disease progression within 20–24 months [2, 3, 7]. Further research is urgently needed to reveal the mechanism of GIST progression and explore novel therapeutic targets to combat imatinib resistance.

Currently, bioinformatic tools are being used to evaluate the molecular signatures associated with progression and clinical outcomes in several types of malignancies [8–10]. In this study, the GSE136755 and GSE47911 datasets were downloaded from the Gene Expression Omnibus (GEO) and used to evaluate the potential target genes involved in GIST progression. Among several hub genes in the GSE136755 dataset, AURKA was considered a key hub gene in GIST progression. Gene set enrichment analysis (GSEA) based on the GSE47911 dataset indicated that AURKA promotes GIST progression by regulating cell cycle processes. Subsequent clinical data analyses demonstrated the value of AURKA as a prognostic factor for GISTs. Furthermore, overexpression of AURKA was experimentally demonstrated to significantly promote GIST/T1 cell proliferation, inhibit apoptosis, and enhance their resistance to imatinib.

## Materials and methods

### Data acquisition and differentially expressed gene (DEG) identification

The GSE136755 dataset was downloaded from the Gene Expression Omnibus (GEO) (https://www.ncbi.nlm.nih.gov/geo/) [11]. GSE136755 is based on the GPL17077 platform (Agilent-039494 SurePrint G3 Human GE v2 8 × 60 K Microarray 039381) and includes clinicopathological information for 65 human GIST tumor samples without preoperative imatinib treatment. Primary GISTs with KIT mutations were selected to screen the differentially expressed genes (DEGs). Advanced GIST samples were defined as samples from patients with high-risk GISTs (16 samples), while nonadvanced GIST samples were defined as samples from patients with low-risk and very low-risk GISTs (31 samples). Intermediate-risk GISTs were not included because they do not have clear distinct biological behaviors in comparison with high-risk and low-risk GISTs. GEO2R, an R-associated web tool from the National Center for Biotechnology Information, was used to screen the DEGs between advanced and nonadvanced GISTs [12]. The DEGs were identified using the cutoff values of |log2FoldChange| > 1 and adju. p < 0.05. For hierarchical clustering analysis in Morpheus (https://software.broadinstitute.org/morpheus), the DEGs were downloaded in text format.

### Functional enrichment analysis of DEGs

The Database for Annotation, Visualization, and Integrated Discovery (DAVID) (http://david.ncifcrf.gov, version 6.8) is a web-based bioinformatics resource that is used to extract genes functional annotation information [13]. Gene ontology (GO) is a major bioinformatics tool for gene annotation and analysis [14, 15]. The Kyoto Encyclopedia of Genes and Genomes (KEGG) is a popular database for the analysis of advanced gene functions and potential signaling pathways in large-scale molecular data [16, 17]. DAVID was used to perform GO and KEGG enrichment analyses of DEGs. The cutoff criterion was a false discovery rate (FDR) < 0.05.

### Protein–protein interaction (PPI) network and module analysis

The Search Tool for the Retrieval Interacting Genes (STRING) (http://string-db.org) is an online database that is used to identify interactions among DEGs [18, 19]. A confidence score ≥ 0.7 was set for conducting the PPI network. Cytoscape (version 3.7.1) is an open-source bioinformatics software platform that is used for visualizing the PPI network and for further analyses [20, 21]. The Molecular Complex Detection (MCODE) plugin in Cytoscape was used to identify significant modules based on the PPI network topology. The criteria were degree cutoff = 2, node score cutoff = 0.2, K-core = 2, and max. depth = 100. The plug-in app ClueGO was used to analyze and visualize the biological processes and pathways in significant modules.

### Hub gene identification and analysis

The cytoHubba plugin in Cytoscape was used to identify hub genes based on the degree and bottleneck algorithms. The Oncomine database is an online platform that computes gene expression signatures, clusters, and gene-set modules [22]. The Gene Expression Profiling Interactive Analysis (GEPIA) database is a newly developed web server for cancer, normal gene expression profiling and interactive analysis [23]. CytoHubba was used to identify the hub genes whose expression patterns have been evaluated in common human malignancies using the Oncomine and GEPIA databases.

Clinicopathological features and KIT/PDGFRα mutation types were extracted from the GSE136755 dataset and from the raw data provided by Lagarde et al. [24]. Correlations between key gene expression patterns and clinicopathological features as well as KIT/PDGFRα mutation types were statistically determined, with P < 0.05 set as the threshold for statistical significance.

### Gene set enrichment analysis (GSEA)

GSEA is a computational method that determines whether an a priori defined gene set shows statistically significant and concordant differences between two biological states [25, 26]. GSEA computes biological information from different perspectives and further elucidates relevant biological events. The GSE47911 dataset, based on the GPL6480 platform (Agilent-014850 Whole Human Genome Microarray 4 × 44 K G4112F), was also downloaded from the GEO database. This dataset has 15 gastric GIST samples [27]. Based on the key gene expression level, GIST samples were divided into two groups, after which GSEA was subsequently performed. Annotated gene sets (c2. cp.kegg.v7.2.symbols.gmt [Curated], c2.cp.ractome.v7.2.symbols.gmt [Curated], and c5.bp.v7.2.symbols.gmt. [Gene Oncology]) were chosen as the reference gene sets. Gene size > 20, FDR < 0.05 and normalized enrichment score (NES) > 2.00 were set as the cutoff criteria.

### Immunohistochemistry and survival analysis

Between 2001 and 2015, a total of 49 patients admitted to the First Affiliated Hospital of Zhejiang University (Zhejiang Province, China) who were diagnosed with GISTs were enrolled in this study. The GIST patients who had incomplete resection, neoadjuvant or adjuvant imatinib treatment, or a family history of GIST, were excluded in this study. Clinical stratification of GISTs was based on the modified NIH criteria [4]. Paraffin-embedded GIST samples were obtained from the study participants and analyzed by immunohistochemical (IHC) staining and were used for the survival analysis. All GIST tissue samples were provided by the Department of Pathology within the First Affiliated Hospital of Zhejiang University. IHC staining was performed as previously described [28]. Briefly, tissue sections were incubated at 4 °C overnight with anti-human AURKA rabbit polyclonal antibody diluted 1:500 (NOVUS Biologicals, USA). A total of five adjacent fields using 400× magnification in areas with the highest density of positive staining were scored according to the summation of the percentage of staining intensity. The immunostaining percentage was defined as 0 (< 5%), 1 (< 20%), 2 (20–50%), and 3 (> 50%). Staining intensity was defined from 0 (no staining) to 3 (strongest staining). The maximum score of IHC staining was 6, in which > 50% of the cells had the strongest staining intensity. Staining scores lower than the mean value were considered low expression, while scores higher than the mean value were considered high expression. The follow-up time for all the patients was calculated from the date of surgery to the date of disease recurrence or last visit. The use of human tumor samples and clinical data in this study was approved by the Ethical Committee of the First Affiliated Hospital of Zhejiang University. Study participants were required to sign written informed consent before enrollment.

### Cell culture

The GIST cell line (GIST/T1) donated by Prof. Wenbin Chen (Zhejiang University, Hangzhou, China) was obtained from the Cell Bank of the Type Culture Collection of the Chinese Academy of Sciences (Shanghai, China). Cells were maintained in RPMI-1640 medium (GIBCO) supplemented with 10% heat-inactivated fetal bovine serum (GIBCO) in a humidified atmosphere with 5% CO_2_ at 37 °C.

### Construction and transfection of lentiviral vectors for AURKA overexpression

The DNA fragment encoding the AURKA sequence was synthesized and inserted into the lentivirus expression vector pLVX-IRES-tdtomato (TaKaRa, China). The resulting vector was identified as pLVX-AURKA-IRES-tdtomato. Lentiviral plasmids were transfected into HEK 293T cells with psPAX2 and pMD2. G plasmids at a ratio of 4:3:1 using Lipofectamine 2000 (Invitrogen, USA). Then, 48 h after transfection, the virus was isolated. GIST/T1 cells were infected with lentivirus for 48 h, and the transfection efficiency was measured by PCR and western blotting.

### RNA extraction and real-time quantitative PCR (RT-qPCR)

Total RNA was extracted using TRIzol reagent (Generay Biotech, China) according to the manufacturer’s instructions. The extracted RNA was then treated with RQ1 RNase-Free DNase (Promega, USA). Then, reverse transcription was performed using PrimeScriptTM RT Master Mix (Takara, China) according to the manufacturer’s instructions. RT-qPCR analysis was performed to measure the expression levels of AURKA using the CFX Connect Real-Time System (BIO-RAD, USA) with the SuperReal PreMix Color SYBR Green kit (Tiangen, China). The primer sets used for RT-qPCR are shown in Table 1. Gene expression levels were normalized against the internal control using the 2^−ΔΔCT^ method.

**Table 1:** Sequences of primers used for quantitative real-time PCR analysis

Gene	Forward (5′–3′)	Reverse (5′–3′)
Homo *AURKA*	TGGGTGGTCAGTACATGCTC	TGCATCCGACCTTCAATCATTTC
Homo *ACTB*	AGCGAGCATCCCCCAAAGTT	GGGCACGAAGGCTCATCATT

### Western blotting

Protein lysates from each sample were separated by 10% sodium dodecyl sulfate-polyacrylamide gel electrophoresis (SDS-PAGE) and transferred to a polyvinylidene difluoride (PVDF) membrane (Merck Millipore, USA). The membrane was blocked with 5% nonfat milk or 5% BSA in TBST (tris-buffered saline with 0.1% Tween 20) for 1 h at room temperature. Then, the membrane was incubated with a primary antibody against AURKA (1:1000 dilution, NOVUS Biologicals, USA) overnight at 4 °C, washed three times using 0.1% TBST buffer for 30 min, probed with goat anti-rabbit IgG-HRP secondary antibody (1:2000 dilution; Santa Cruz, USA), and washed three times using 0.1% TBST buffer for 30 min. The HRP-conjugated secondary antibody was detected and visualized using an enhanced chemiluminescence detection system (GE Healthcare, USA). Band intensity was quantified by densitometry using ImageJ software (version 1.49; National Institutes of Health, USA) [29].

### Proliferation assay

Cell proliferation was assessed by the CCK-8 assay (Tongren Chemical Society, Japan). Briefly, 1 × 10^5^ cells per well were seeded in 96-well plates and incubated in a 5% CO_2_ atmosphere at 37 °C for 24 h. Imatinib (3 µM) was then added to the culture medium for 48 h to evaluate the effect of AURKA on imatinib resistance. The medium was discarded, replaced with serum-free medium and CCK8 (10 µl), and incubated for 2 h. The Biokinetics Reader (MD corporate, USA) was used to detect absorbance at 450 nm.

### Apoptosis assay

Cell apoptosis was assessed using the Annexin V-APC/7-AAD apoptosis kit (MultiSciences, China) according to the manufacturer’s instructions. Briefly, 3 × 10^5^ cells per well were seeded in 6-well plates and incubated in a 5% CO_2_ atmosphere at 37 °C for 24 h. Imatinib (3 µM) or solvent was added to the culture medium for 48 h, after which the cells were collected. After being washed twice in PBS at 4 °C, cells were resuspended in binding buffer (500 µl). Annexin V-APC (5 µl) and 7-AAD (10 µl) were added to the suspension and the mixture was incubated for 5 min at 4 °C. The apoptosis index was examined by flow cytometry (ACCURI C6; BD, USA).

### Statistical analysis

All statistical analyses were performed using SPSS 25.0 software (SPSS Inc., USA). Descriptive data were expressed as the mean ± SD. Comparison of more than two mean values was performed by one-way analysis of variance (ANOVA), while Student’s *t*-test was used to compare two mean values. Kaplan–Meier analysis was performed to establish disease-free survival (DFS) curves, while the log-rank test was used for survival curve comparison. The Cox proportional hazards model was then used to perform multivariable analysis, while the forward likelihood ratio method was used to identify independent variables. P < 0.05 was set as the threshold for statistical significance.

## Results

### Identification of differentially expressed genes (DEGs)

**Fig. 1:**
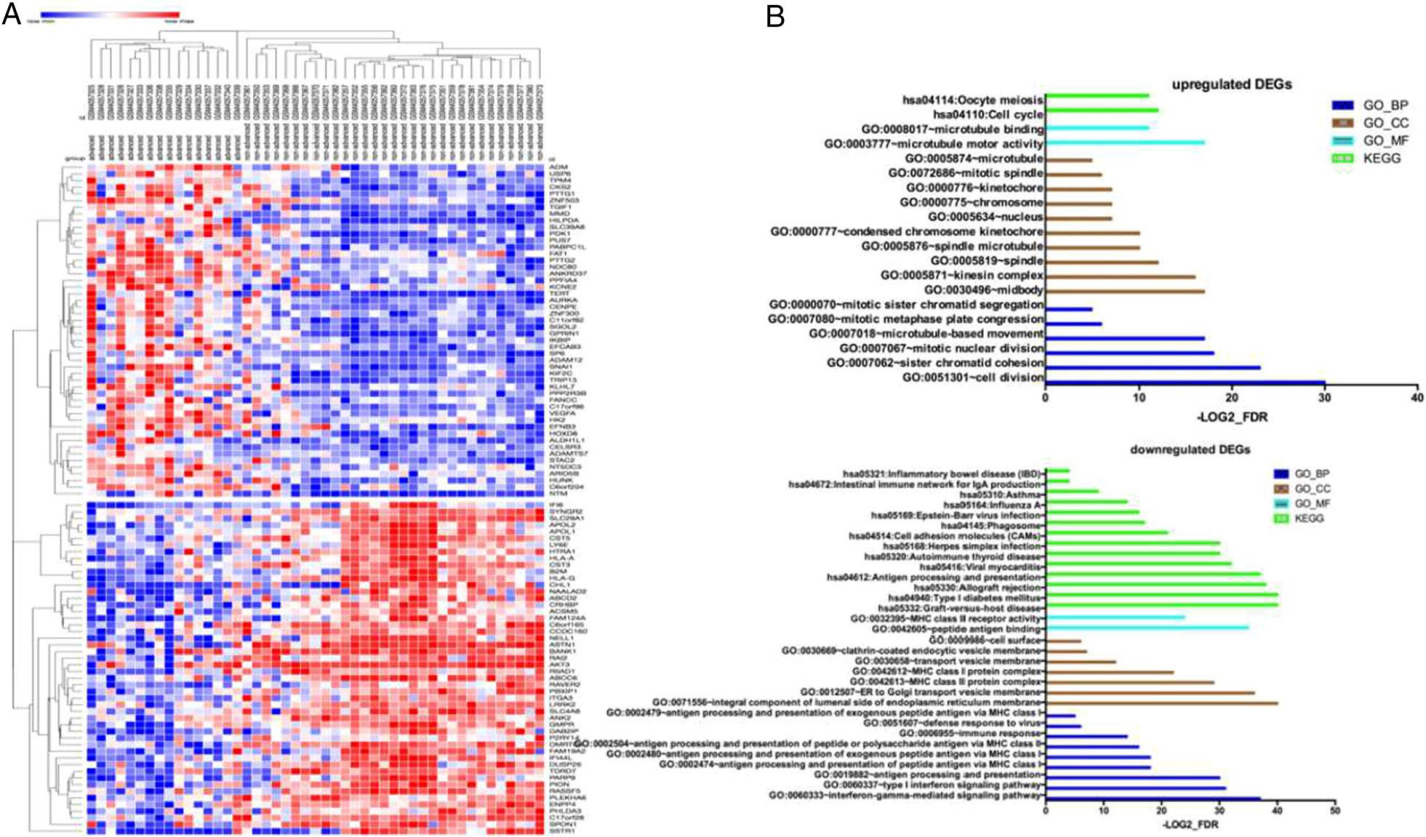
Screening of differentially expressed genes (DEGs) from the GSE136755 dataset. **A** Heat map generated based on the top 50 up-and downregulated DEGs between samples from advanced and nonadvanced GISTs. **B** Functional annotation of DEGs by GO and KEGG enrichment analyses

A total of 65 human GIST samples without preoperative imatinib treatment were included in the GSE13675 dataset. A cohort of 47 samples with KIT mutations, consisting of 16 patients with advanced (high-risk) GIST and 31 with nonadvanced (low-risk and very low-risk) GIST, was used to screen the DEGs using GEO2R. A total of 606 genes (244 with upregulated expression and 362 with downregulated expression) were identified using the cutoff criteria of adj. p < 0.05 and log2FoldChange > 1. The top 50 genes with up-and downregulated expression are presented as a heatmap (Fig. 1A, Additional file 1).

### Functional annotation of DEGs

To evaluate the biological clustering of DEGs, GO and KEGG analyses for the up- and downregulated DEGs were performed using DAVID. Based on GO analysis, upregulated DEGs were found to be significantly enriched in cell division, sister chromatid cohesion, mitotic nuclear division, microtubule-based movement, and mitotic metaphase plate congression. Cellular components (CCs) of the upregulated DEGs were significantly enriched in the midbody, kinesin complex, spindle, spindle microtubule, and condensed chromosome kinetochore. The molecular function (MF) of the upregulated DEGs was significantly enriched in microtubule motor activity and microtubule binding. KEGG analysis showed that the upregulated DEGs were mainly involved in the cell cycle and oocyte meiosis. GO analysis revealed that the downregulated DEGs were significantly enriched in the interferon-gamma-mediated signaling pathway, type I interferon signaling pathway, and antigen processing and presentation of antigens. The CC of the downregulated DEGs was significantly enriched in the integral component of the luminal side of the endoplasmic reticulum membrane and ER to Golgi transport vesicle membrane, while the MF of the downregulated DEGs was significantly enriched in peptide antigen binding and MHC class II receptor activity. KEGG analysis further revealed that the downregulated DEGs were mainly involved in graft-versus-host disease, type I diabetes mellitus, allograft rejection, antigen processing and presentation, and viral myocarditis. These results are presented in Fig. 1B (Additional file 2).

### Module analysis through the PPI network of DEGs

**Fig. 2:**
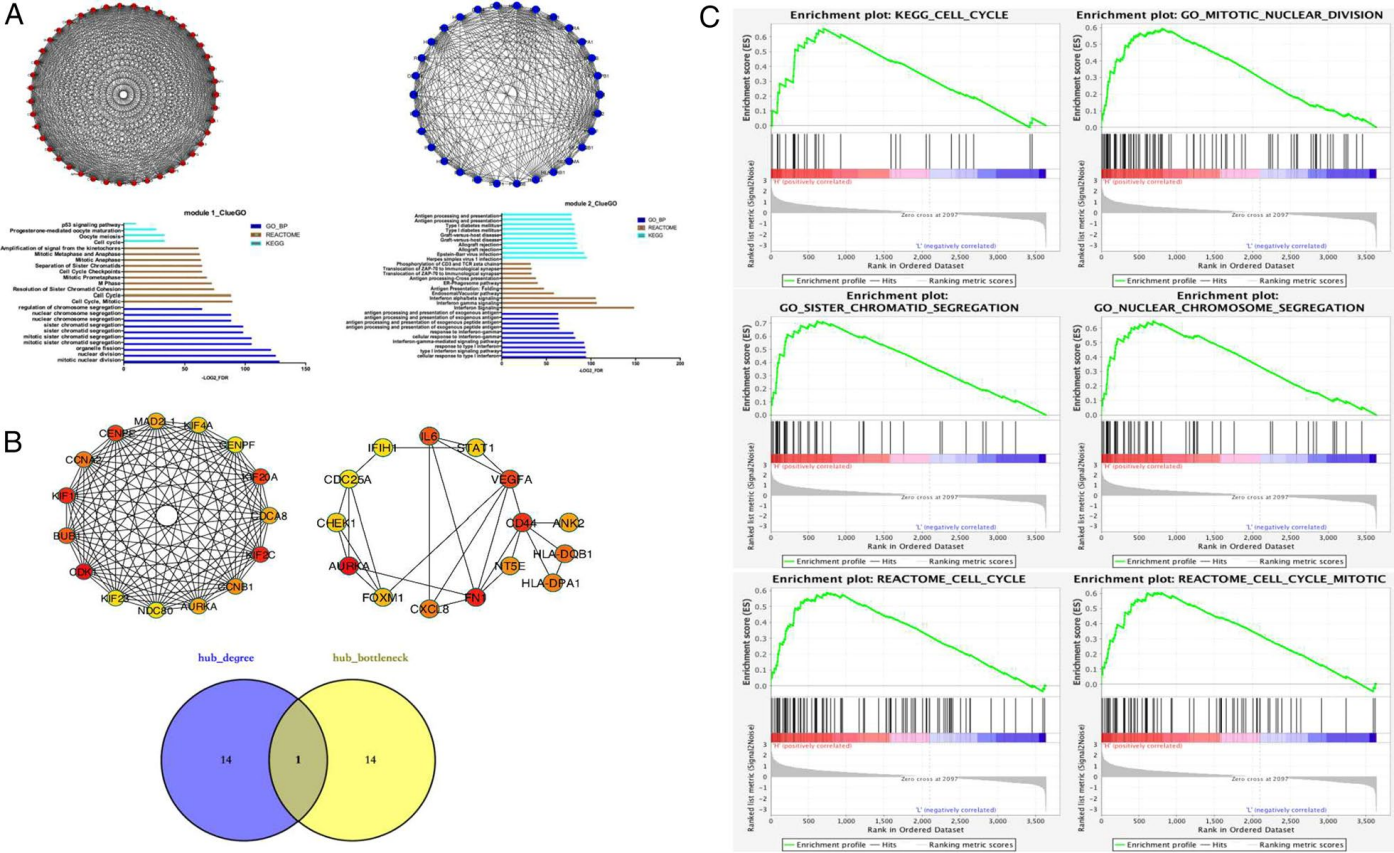
Identification of AURKA as a key hub gene in GISTs. **A**, **B** Based on dataset GSE136755, the most significant modules based on the topology in the protein–protein interaction (PPI) network were identified using Molecular Complex Detection (MCODE) of Cytoscape. Module 1 contains upregulated DEGs (red color), while module 2 contains downregulated DEGs (blue color). Functional annotation of modules 1 and 2 was determined by ClueGO. Hub genes were identified using the cytoHubba plugin in Cytoscape based on the degree and bottleneck algorithms. The key hub gene (AURKA) was obtained by taking the intersection of the two groups of hub genes. **C** Based on the GSE47911 dataset, gene set enrichment analysis (GSEA) results showed cell cycle-related gene enrichment in GISTs with elevated AURKA expression

To clarify the DEGs functionally, STRING was used to construct a PPI network, which was composed of 603 nodes and 1768 edges. The PPI enrichment p-value was < 1.0E−16. The PPI network was visualized by Cytoscape and further analyzed by the MCODE plugin. The two most significant modules (module 1 and module 2) were identified and analyzed using GO, KEGG and REACTOME annotations to infer their biological functions. Module 1 (MCODE score = 36.667) was mainly involved in cell cycle-related biological processes and signaling pathways, while module 2 (MCODE score = 18.759) was mainly involved in immunological processes and signaling pathways. These results are presented in Fig. 2A (Additional file 2).

### Identification of hub genes

To reveal the crucial genes underlying the regulation of GIST progression, we filtered hub genes among DEGs using the cytoHubba plugin of Cytoscape. Two algorithms, degree and bottleneck, were applied to weight the DEGs. The degree algorithm calculates the relevance and abundance of genes, while the bottleneck algorithm evaluates key gene positions in an entire regulatory network. According to the degree algorithm, the top 15 hub genes were CDK1, KIF11, KIF2C, CENPE, KIF20A, BUB1, CCNA2, CCNB1, AURKA, MAD2L1, CDCA8, KIF4A, CENPF, NDC80, and KIF23, with scores ranging from 58 to 48. According to the bottleneck algorithm, the top 15 hub genes were AURKA, FN1, CD44, VEGFA, IL6, HLA-DQB1, HLA-DPA1, CXCL8, NT5E, ANK2, FOXM1, CHEK1, STAT1, CDC25A, and IFIH1, with scores ranging from 64 to 8. A Venn diagram was used to identify the intersection of the key hub genes between the two hub gene cohorts. The results showed that AURKA was the only overlapping hub gene (Fig. 2B).

### Gene set enrichment analysis (GSEA)

There was a total of 15 human gastric GIST samples in the GSE47911 dataset, comprised of 6 high-risk patients, 1 intermediate-risk patient, 3 low-risk patients, and 5 very low-risk patients. To further verify significant biological processes associated with AURKA expression, GSE47911 gene profiles were divided into two groups, after which GSEA was performed based on the AURKA expression level. Samples with the highest (25%, 4 samples) and lowest (25%, 4 samples) expression levels were selected for further analysis using GSEA. Cell cycle-related gene sets were associated with elevated AURKA expression (Fig. 2C, Additional file 3).

### Correlation between AURKA expression and the clinicopathological features of GISTs

**Fig. 3:**
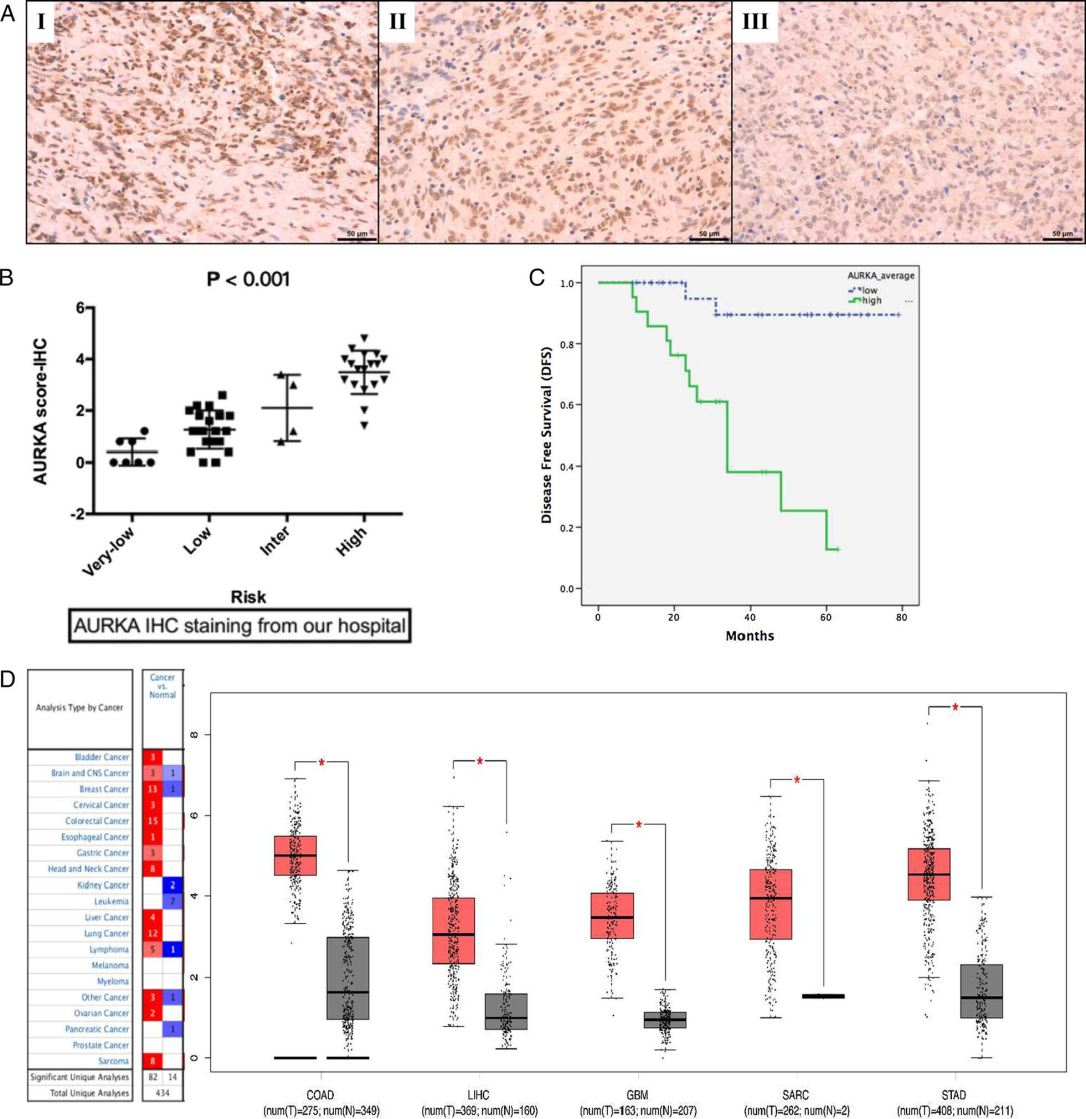
Expression patterns of AURKA in GISTs and other human malignancies. **A** Immunohistochemical (IHC) staining of GIST specimens. I: strong staining, II: intermediate staining, III: weak staining. **B** IHC scores of AURKA varied across different GIST risk groups, with high-risk GISTs showing the highest expression of AURKA. **C** Patients with elevated AURKA expression levels exhibited poorer DFS than those with low AURKA expression levels. **D** The Oncomine and GEPIA databases showed a significant upregulation of AURKA expression in most human malignancies compared to normal tissues

**Fig. 4:**
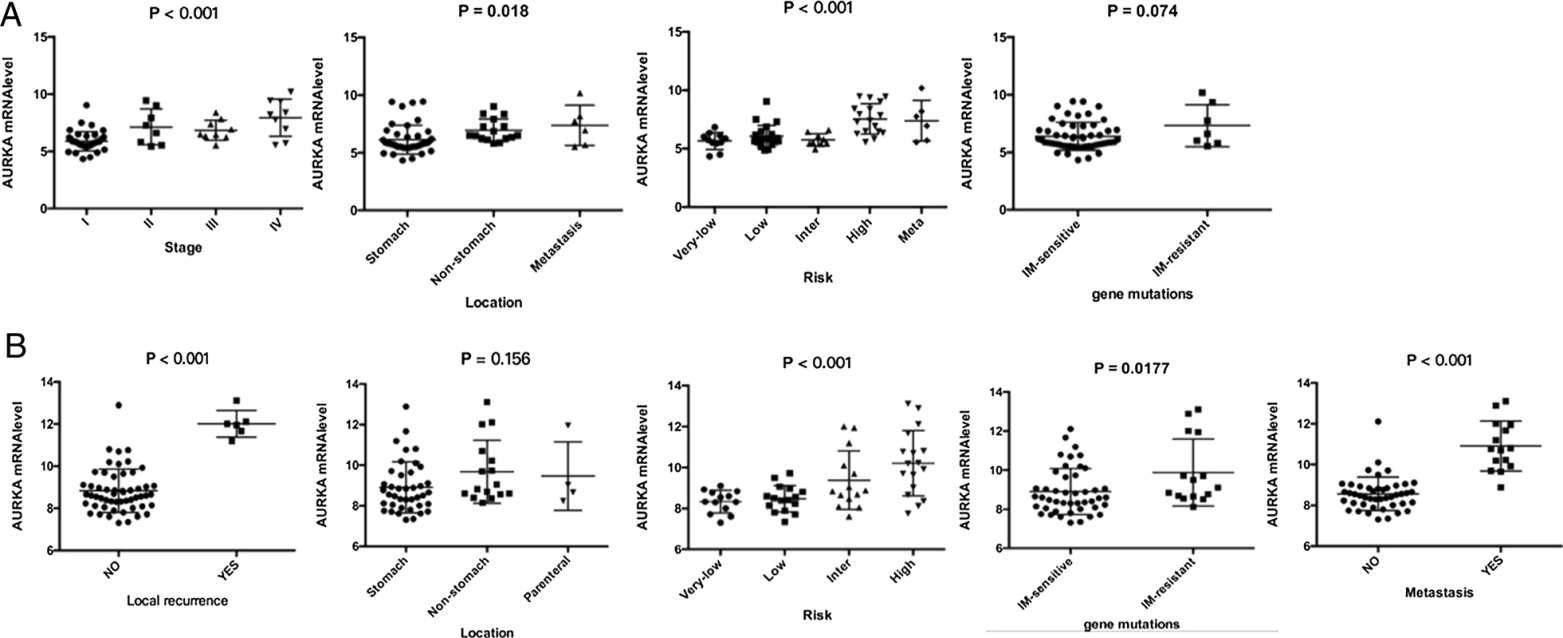
Expression profiles of AURKA in different GIST cohorts. **A** Based on data from the GSE136755 dataset, AURKA expression was significantly associated with tumor location, stage and risk stratification, while there was no obvious correlation between AURKA expression and imatinib-related gene mutations. **B** Analysis of the raw data from Lagarde et al. (PMID: 22167411) revealed that AURKA expression was significantly associated with risk stratification and imatinib-related gene mutations. There is also an obvious correlation between AURKA expression and clinical outcomes, such as local recurrence and metastasis. There were no significant differences in AURKA expression levels among gastric, nongastric and parenteral GISTs

To evaluate the clinical significance of AURKA expression in GISTs, AURKA expression levels in 49 GIST tissues were assessed by IHC staining (Fig. 3A). The correlations between AURKA expression and clinicopathological features (age, sex, location and risk stratification) were determined (Table 2, Additional file 4). AURKA expression was closely associated with tumor risk stratification (Fig. 3B; P < 0.001). The clinical significance of AURKA expression in GISTs was also evaluated using the data from GSE136755 and the raw data provided by Lagarde et al. [24] (Tables 3 and 4, and Fig. 4, Additional file 5). Findings from the GSE136755 dataset analysis revealed significant associations between AURKA expression and tumor risk stratification (P < 0.001) as well as tumor stage (P < 0.001) and the analyses of the raw data from Lagarde et al. [24] also showed a significant association between AURKA expression and tumor risk stratification (P < 0.001) as well as tumor recurrence (P < 0.001) and metastasis (P < 0.001). However, apart from GSE136755, which revealed a significant association between AURKA expression and tumor location (P = 0.018), the data provided by Lagarde et al. did not establish a significant association between AURKA expression and tumor location (P = 0.156).

**Table 2:** Correlation between AURKA expression level and clinical features in 49 GIST patients

Parameter	Characteristic	No. patients (%)	AURKA score	Statistics	P value
Gender	Female	23 (46.9)	1.93 ± 1.35	t = − 0.430	0.669
	Male	26 (53.1)	2.12 ± 1.52		
Age	< 57	24 (49.0)	2.05 ± 1.52	t = − 0.121	0.904
	≥ 57	25 (51.0)	2.00 ± 1.36		
Location	Stomach	14 (28.6)	1.74 ± 1.30	F = 1.321	0.277
	Small intestine	12 (24.5)	1.68 ± 1.30		
	Large intestine	23 (46.9)	2.37 ± 1.53		
Risk*	High	18 (36.7)	2.02 ± 1.43	F = 35.449	< 0.001
	Intermediate	4 (8.2)	2.1 ± 1.29		
	Low	20 (40.8)	1.26 ± 0.74		
	Very low	7 (14.3)	0.40 ± 0.52		

*P < 0.05 was considered statistically significant

**Table 3:** Correlation between *AURKA* intensity and clinical features based on GSE136755

Parameter	Characteristic	No. patients (%)	AURKA intensity	Statistics	P value
Gender	Female	26 (40.0)	6.19 ± 0.97	t = − 1.458	0.150
	Male	39 (60.0)	6.63 ± 1.47		
Age	< 68	31 (47.7)	6.28 ± 1.17	t = 1.022	0.311
	≥ 68	34 (52.3)	6.62 ± 1.41		
Location*	Stomach	43 (66.2)	6.14 ± 1.25	F = 4.306	0.018
	Non-stomach	16 (24.6)	6.95 ± 0.99		
	Metastasis	6 (9.2)	7.39 ± 1.74		
Risk*	High	17 (26.2)	7.54 ± 1.30	F = 11.898	< 0.001
	Intermediate	9 (13.8)	5.75 ± 0.54		
	Low	22 (33.8)	6.06 ± 0.94		
	Very low	11 (16.9)	5.66 ± 0.74		
Stage*	IV	9 (13.8)	7.94 ± 1.63	F = 10.686	< 0.001
	III	9 (13.8)	6.85 ± 0.89		
	II	8 (12.3)	7.13 ± 1.55		
	I	39 (60.0)	5.89 ± 0.84		
Mutation	Sensitive	56 (86.2)	6.39 ± 1.21	F = 2.541	0.087
	Resistance	7 (10.8)	7.33 ± 1.84		
	N/A	2 (3.1)	5.29 ± 0.20		
Mutation	Sensitive	56 (88.9)	6.39 ± 1.21	t = 1.814	0.075
	Resistance	7 (11.1)	7.33 ± 1.84		

*P < 0.05 was considered statistically significant

**Table 4:** Correlation between *AURKA* intensity and clinical features based on the published raw data

Parameter	Characteristic	No. patients (%)	AURKA intensity	Statistics	P value
Location	Stomach	40 (66.7)	8.91 ± 1.25	F = 1.923	0.156
	Non-stomach	16 (26.7)	9.64 ± 1.55		
	Parenteral	4 (6.7)	9.47 ± 1.69		
AFIP risk*	High	17 (28.3)	10.2 ± 1.59	F = 8.686	< 0.001
	Intermediate	14 (23.3)	9.38 ± 1.43		
	Low	16 (26.7)	8.48 ± 0.64		
	Very low	13 (21.7)	8.34 ± 0.55		
Local recurrence*	No	54 (90.0)	8.83 ± 1.03	t = 7.377	< 0.001
	Yes	6 (10.0)	12.0 ± 0.63		
Metastasis*	No	45 (75.0)	8.56 ± 0.82	t = 6.921	< 0.001
	Yes	15 (25.0)	10.9 ± 1.22		
Mutation*	Sensitive	45 (75.0)	8.91 ± 0.18	t = 2.442	0.018
	Resistance	15 (25.0)	9.88 ± 0.44		

*P < 0.05 was considered statistically significant

To determine the prognostic value of AURKA expression in GISTs, Kaplan-Meier survival analysis was performed. The range of observation time was 9–79 months. As shown in Fig. 3C, patients with GIST with elevated AURKA expression exhibited poorer DFS than those with low AURKA expression levels (43.25 ± 6.94 months vs. 98.48 ± 3.44 months, P < 0.001). We further included the statistically significant variables (tumor size, mitotic index, risk stratification, and AURKA score) into Cox regression model. The Cox proportional hazards model showed that AURKA could be used as an independent prognostic marker for GISTs (P = 0.002) and the hazard ratio (HR) was 0.087.

Gene mutation types can predict the responsiveness of GISTs to imatinib. GISTs with KIT exon 11, PDGFRα exon 12 and PDGFRα exon 14 mutations were considered sensitive to imatinib. GISTs with other mutations, such as KIT exon 9, KIT exon 13, KIT exon 14, KIT exon 17, KIT exon 18, PDGFRα exon 18 and D842V mutations and KIT/PDGFRα wild-type GISTs, were insensitive/resistant to imatinib [1, 3, 30]. In the GSE136755 dataset, 56 samples were from imatinib-sensitive GISTs and 7 were from imatinib-resistant GISTs; there was a weak association between AURKA expression and imatinib-resistant gene mutations (Fig. 4A, P = 0.074). Through analysis of the raw data provided by Lagarde et al. [24], comprising 45 samples from imatinib-sensitive GISTs and 15 from imatinib-resistant GISTs, it was shown that there was a strong association between AURKA expression and imatinib-resistant gene mutations (Fig. 4B, P = 0.018).

### AURKA expression patterns in common human malignancies

To determine whether elevated AURKA expression is common in human digestive malignancies, mRNA expression levels of AURKA in stomach carcinoma, liver hepatocellular carcinoma, and colorectal carcinoma were evaluated using data from the GEPIA database. AURKA expression was found to be significantly upregulated in all the above malignancies compared to normal tissues. Findings from the Oncomine database also indicated that AURKA expression is upregulated in most human malignancies (Fig. 3D).

### AURKA overexpression promotes GIST/T1 cell proliferation and anti-apoptosis

**Fig. 5:**
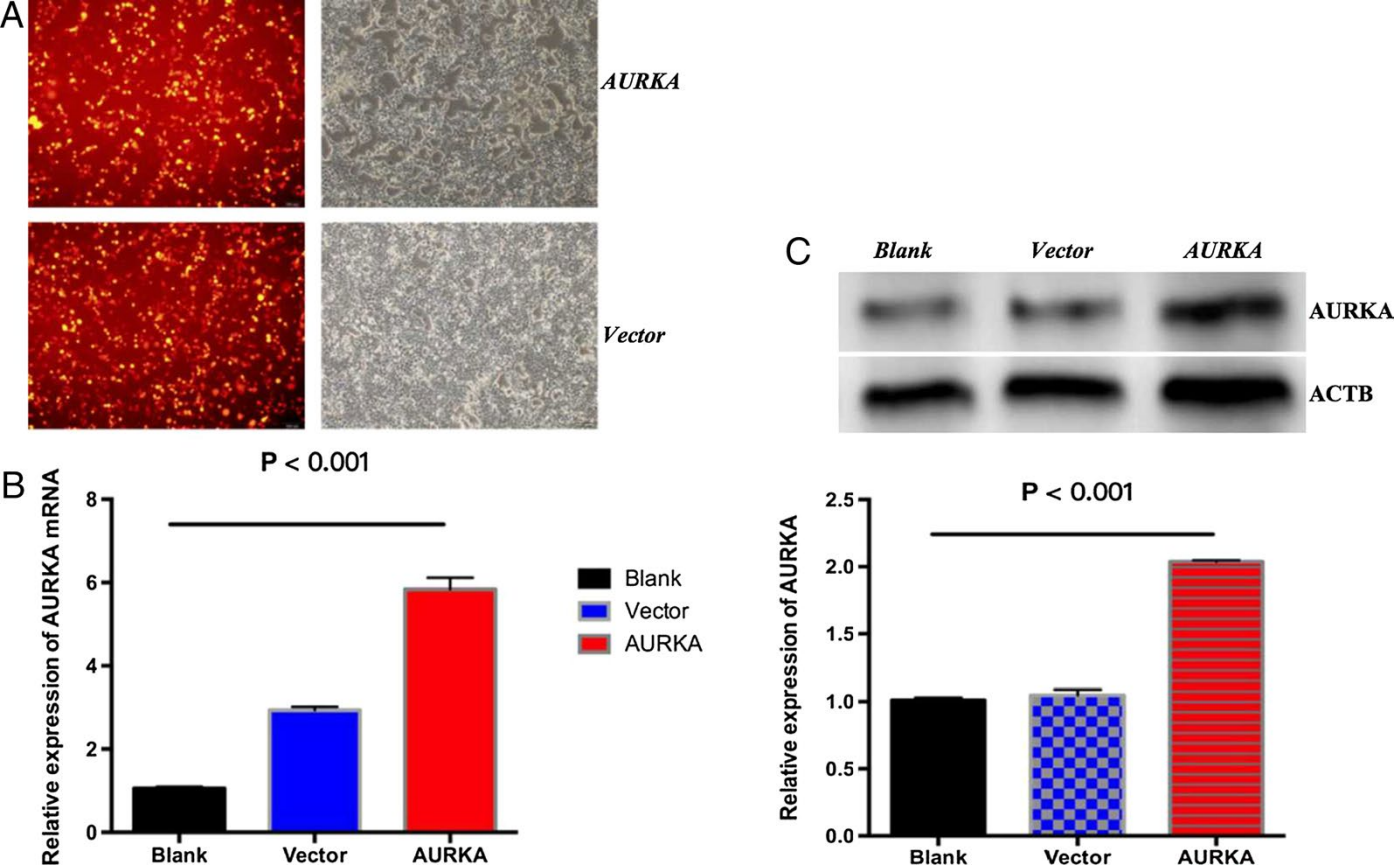
Construction of GIST/T1 cells overexpressing AURKA. **A** Representative images of cells transfected with AURKA plasmids and control plasmids captured with fluorescence and bright field microscopy. **B** mRNA expression levels of AURKA were significantly higher in the *AURKA* group than in the *blank* and *vector* groups. **C** Protein expression levels of AURKA were elevated in the *AURKA* group compared with the *blank* and *vector* groups

To assess the biological effects of AURKA expression in GISTs, AURKA was overexpressed in GIST/T1 cells by transfection with an AURKA-expressing virus; these cells were defined as the AURKA overexpression group (*AURKA* group). Normal GIST/T1 cells (*blank* group) and GIST/T1 cells transfected with vacant plasmids (*vector* group) were considered the control groups. The transfection efficiency was determined by observing the red fluorescence from the tdtomato reporter and quantified by RT-qPCR and western blotting. Figure 5 shows that compared to the *blank* and *vector* groups, AURKA was overexpressed in the *AURKA* group (Additional file 6).

**Fig. 6:**
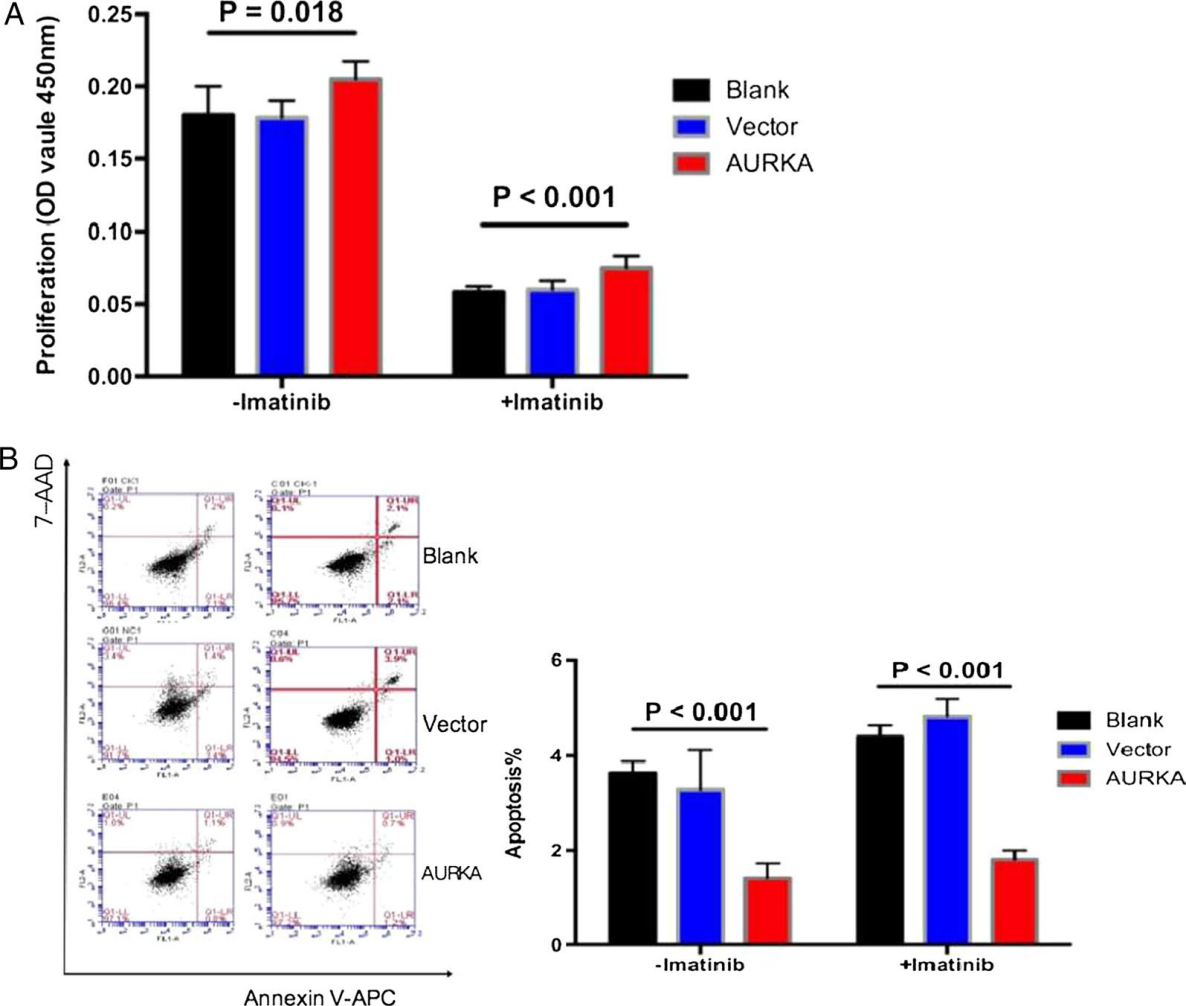
The effect of AURKA overexpression on the aggressive behaviors of GIST cells. **A** AURKA overexpression significantly promoted the proliferation of GIST/T1 cells. **B** Annexin V assay revealed that AURKA overexpression significantly inhibited cell apoptosis and enhanced imatinib resistance in GIST/T1 cells

The CCK-8 assay was performed to assess the effect of AURKA overexpression on cell proliferation. Compared to the *blank* and *vector* groups, the overexpression of AURKA in the *AUKRA* group significantly enhanced GIST/T1 cell proliferation (P = 0.018) (Fig. 6A). Imatinib treatment significantly inhibited cell proliferation in all three groups. However, compared to cells in the control groups, *AUKRA* group cells still showed a relatively higher proliferation rate in the presence of imatinib (P < 0.001, Fig. 6A; Additional file 6).

We also established that AURKA overexpression markedly suppressed the apoptotic process in GIST/T1 cells (P < 0.001, Fig. 6B). A similar result was observed with imatinib administration. Compared to the *blank and vector* groups, AURKA overexpression obviously inhibited cell apoptosis after imatinib administration (P < 0.001, Fig. 6B). The results suggested that AURKA overexpression enhanced the resistance of GIST cells to imatinib (Additional file 6).

## Discussion

GIST is a common mesenchymal malignancy of the human alimentary system. Compared to gastrointestinal carcinomas, GISTs are known to possess unique biological features. For example, lymph node metastasis is not common in GIST and preferentially occurs in patients at a young age [31, 32]. A 1- or 2-cm macroscopic margin may be sufficient to achieve microscopically negative margins [33]. GISTs respond poorly to conventional chemotherapy and radiotherapy [34]. As such, to better understand GIST biological behavior and inform the development of therapeutic strategies, it is important to establish the crucial genes that regulate the malignant behavior of GISTs. Bioinformatics advances have been useful for exploring molecular targets indicating the progression and prognosis of GIST [11, 35, 36].

In this study, gene profiles of 47 GIST samples from the GSE136755 dataset were selected for further analyses. Comparison of the gene profiles between advanced and nonadvanced GISTs generated 244 upregulated DEGs and 362 downregulated DEGs. Functional annotation based on GO and KEGG analyses showed that upregulated DEGs were mainly enriched in cell cycle-related biological processes and signaling pathways, while the downregulated DEGs were mainly enriched in immune-related biological processes and signaling pathways. The STRING database and Cytoscape software were used for further exploration of the DEGs. Two important modules were extracted and visualized. Module 1 consisted of upregulated DEGs and was mainly involved in cell cycle-related biological processes and signaling pathways, while module 2 consisted of downregulated DEGs and was mainly involved in immunological processes and signaling pathways. This indicates that the difference in gene profiles between advanced and nonadvanced GISTs is mainly reflected in the cell cycle and tumor immunity.

Based on the degree and bottleneck algorithms, the cytoHubba plugin in Cytoscape software was used to screen for novel key genes associated with GIST progression. The degree algorithm calculates the relevance and abundance of genes, while the bottleneck algorithm evaluates key gene positions in an entire regulatory network. In this study, a significant key gene, AURKA, was identified using a Venn diagram. AURKA is a protein-coding gene that encodes a cell cycle-regulated kinase involved in microtubule formation and/or stabilization at the spindle pole during chromosomal segregation. It has been documented that AURKA promotes tumor progression by enhancing cell cycle progression, cell survival, genomic instability, epithelial-mesenchymal transition (EMT) and stem-like properties of cancer cells [37]. In most solid tumors, AURKA regulates cell cycle checkpoints and promotes the cell cycle process [37]. GSEA based on GSE47911 gene profiles further validated the association between AURKA overexpression and cell cycle progression in GISTs.

To confirm the importance of AURKA expression in GISTs, we performed IHC staining to establish the associations between AURKA expression and the clinicopathological characteristics of the 49 enrolled patients with GIST. In advanced GISTs, the expression level of AURKA was found to be elevated. This result is consistent with the analyses of the data provided by GSE136755 and Lagarde et al. [24]. Survival analysis further showed that AURKA overexpression was a potential independent prognostic factor for patients with GIST. Furthermore, a series of in vitro experiments demonstrated that overexpressing AUKRA in GIST cells promoted cell proliferation and was antiapoptotic, indicating enhanced malignancy. These findings validated the results of the bioinformatics analyses.

Drug resistance is a major obstacle in cancer chemotherapy and greatly affects a patient’s prognosis. Adjuvant imatinib has been widely used as a first-line therapeutic option for patients with advanced GIST [38, 39]. However, the prevalence of imatinib resistance has increased in recent years. By analyzing the raw data provided by Lagarde et al. [24], a significant relationship was found between AURKA overexpression and gene mutations causing imatinib resistance. The GSE136755 dataset showed a similar result, but the result was not significant, which could be attributable to the small sample size. In addition, in vitro experiments showed that AURKA overexpression enhanced the resistance of GIST cells to imatinib by promoting cell proliferation and inhibiting cell apoptosis.

The role of AURKA overexpression in tumor progression has been reported in a variety of human malignancies. AURKA phosphorylates RPS6KB1 and promotes cell proliferation and anti-apoptosis [40]. AURKA also stabilizes the transcription factor N-MYC, thereby promoting G1/S cell cycle transition and tumor cell proliferation [41]. Pharmacological inhibition of AURKA promotes the chemosensitivity of cervical cancer cells [42]. Compounds targeting AURKA, particularly alisertib, have been extensively studied in preclinical models, where they have shown synergistic effects with other targeted therapies, leading to tumor regression in a variety of cancer models [43]. Yeh et al. confirmed the contribution of the AURKA inhibitor MLN8237 to the suppression of metastatic GISTs [44]. Findings from the GEPIA and Oncomine databases also supported the contribution of AURKA overexpression to tumorigenesis.

This study had the following limitations. First, the case number in each GIST cohort was not large. Therefore, we made up for this deficiency to some extent by incorporating different GIST cohorts for comprehensive analysis. Second, this study is a preliminary exploration and certification of AURKA as a therapeutic target. We did not carry out experiments to investigate the potential of AURKA-targeted therapy. Further validation based on various in vitro and in vivo experiments is required.

## Conclusions

In conclusion, our findings demonstrated a significant overexpression of AURKA in advanced GISTs by bioinformatics analyses, which predicts poor patient prognosis. Overexpression of AURKA was experimentally demonstrated to promote the proliferation of GIST cells and inhibit GIST cell apoptosis, which contributes to imatinib resistance, implying that AURKA is a potential therapeutic target for GISTs.

## Supplementary Information

**Additional file 1.** The expression matrix of the top 50 up-regulated and down-regulated DEGs.

**Additional file 2.** Functional annotation of DEGs based on GSE136755 dataset.

**Additional file 3.** GSEA based on GSE47911 dataset.

**Additional file 4.** Clinicopathological features, AURKA expression and follow-up of GIST patients.

**Additional file 5.** Characteristics of GIST samples in GSE136755 and raw data provided by Lagarde *et al*. (PMID:22167411).

**Additional file 6.** Raw data from in vitro experiments.

## Data Availability

All data and materials generated and analyzed during this study are included in the main paper.
