# Supplementary material for: Aurora kinase A (AURKA) promotes the progression and imatinib resistance of advanced gastrointestinal stromal tumors
Source: Cancer Cell Int. 2021 Jul 31;21:407. doi: 10.1186/s12935-021-02111-7 (PMC8325869; doi:10.1186/s12935-021-02111-7)
Supplement: Supplementary file 5 — Additional file 5. Characteristics of GIST samples in GSE136755 and raw data provided by Lagarde et al. (PMID:22167411). [file 12935_2021_2111_MOESM5_ESM.docx]

Characteristics of GSE136755 samples downloaded from GEO

| Accession | Age | Gender | Primary tumor site | Metastatic tumor site | Risk | Tumor stage | Kit/pdgfra mutation type | Kit/pdgfra mutation site |
| --- | --- | --- | --- | --- | --- | --- | --- | --- |
| GSM4057318 | 45 | M | duodenum | liver | --- | IV | KIT | E11: p.Y570_L576del |
| GSM4057319 | 68 | M | duodenum | liver | --- | IV | KIT | E11: p.G565_P573delinsA |
| GSM4057320 | 75 | M | stomach | liver | --- | IV | KIT | E11: p.V559D, E13: p.V654A, E17: p.N822Y |
| GSM4057321 | 74 | M | small intestine | liver | --- | IV | KIT | E9: p.A502_Y503dup |
| GSM4057322 | 71 | M | omentum | liver | --- | IV | PDGFRA | E14: p.K646N, p.N659Y |
| GSM4057323 | 76 | M | stomach | peritoneum | --- | IV | PDGFRA | E18: p.D842V |
| GSM4057324 | 75 | M | stomach | --- | high | IV | PDGFRA | E18: p.D842V |
| GSM4057325 | 66 | F | stomach | --- | high | IV | KIT | E11: p.W557_V559delinsF, E13: p.V654A |
| GSM4057326 | 52 | M | small intestine | --- | high | IV | KIT | E11: p.W557G |
| GSM4057327 | 68 | F | stomach | --- | high | IIIB | KIT | E11: p.K550_K558del |
| GSM4057328 | 45 | M | stomach | --- | high | IIIB | KIT | E11: p.W557_K558del |
| GSM4057329 | 74 | M | duodenum | --- | high | IIIB | KIT | E11: p.W557_K558del |
| GSM4057330 | 78 | M | small intestine | --- | high | IIIB | KIT | E11: p.E554_V560delinsV |
| GSM4057331 | 71 | F | colon (rectum) | --- | high | IIIB | KIT | E11: p.W557_K558del |
| GSM4057332 | 55 | F | colon (rectum) | --- | high | IIIB | KIT | E11: p.Q575_D579del, E13: p.K642E |
| GSM4057333 | 86 | M | stomach | --- | high | IIIA | KIT | E11: p.P551_K558delinsQ |
| GSM4057334 | 34 | F | colon (ascending) | --- | high | IIIA | KIT | E11: p.I563_D572del |
| GSM4057335 | 74 | M | stomach | --- | high | IIIA | KIT | E11: p.W557_K558delinsE |
| GSM4057336 | 68 | M | stomach | --- | high | II | KIT | E11: p.W557_E561del |
| GSM4057337 | 79 | M | esophagus | --- | high | II | KIT | E11: p.W557_K558del |
| GSM4057338 | 56 | M | colon (rectum) | --- | high | II | KIT | E11: p.K558_E562del, E14: p.T670I |
| GSM4057339 | 85 | F | stomach | --- | high | II | KIT | E11: p.V559G |
| GSM4057340 | 67 | M | duodenum | --- | high | II | KIT | E11: p.V559G |
| GSM4057341 | 67 | F | stomach | --- | intermediate | II | KIT | E11: p.V560del |
| GSM4057342 | 62 | M | stomach | --- | intermediate | II | PDGFRA | E18: p.D842_M844del |
| GSM4057343 | 63 | M | stomach | --- | intermediate | II | PDGFRA | E18: p.D842V |
| GSM4057344 | 65 | M | stomach | --- | intermediate | IB | KIT | E11: p.W557_V559delinsC |
| GSM4057345 | 75 | F | stomach | --- | intermediate | IB | KIT | E11: p.L576_D579dup |
| GSM4057346 | 90 | F | stomach | --- | intermediate | IB | KIT | E11: p.L576P |
| GSM4057347 | 66 | F | stomach | --- | intermediate | IB | KIT | E11: p.V560D |
| GSM4057348 | 65 | M | stomach | --- | intermediate | IB | KIT | E11: p.V560D |
| GSM4057349 | 74 | F | stomach | --- | intermediate | IB | --- | --- |
| GSM4057350 | 61 | F | stomach | --- | low | IA | KIT | E11: p.W557_K558del |
| GSM4057351 | 60 | M | stomach | --- | low | IA | KIT | E11: p.K558_E562del |
| GSM4057352 | 60 | M | stomach | --- | low | IA | KIT | E11: p.D579del |
| GSM4057353 | 64 | M | stomach | --- | low | IA | KIT | E11: p.W557_K558del |
| GSM4057354 | 71 | F | stomach | --- | low | IA | KIT | E11: p.V569_L576del |
| GSM4057355 | 72 | F | stomach | --- | low | IA | KIT | E11: p.D579del |
| GSM4057356 | 77 | M | stomach | --- | low | IA | KIT | E11: p.V560del |
| GSM4057357 | 74 | F | stomach | --- | low | IA | KIT | E11: p.Y578_D579dup |
| GSM4057358 | 71 | F | stomach | --- | low | IA | KIT | E11: p.L576_P585dup |
| GSM4057359 | 65 | F | stomach | --- | low | IA | KIT | E11: p.V559D |
| GSM4057360 | 80 | M | stomach | --- | low | IA | KIT | E11: p.L576P |
| GSM4057361 | 81 | M | stomach | --- | low | IA | KIT | E11: p.V559D |
| GSM4057362 | 63 | M | stomach | --- | low | IA | KIT | E11: p.V559A |
| GSM4057363 | 73 | M | stomach | --- | low | IA | KIT | E11: p.V559D |
| GSM4057364 | 55 | M | stomach | --- | low | IA | PDGFRA | E12: p.V561D |
| GSM4057365 | 77 | F | stomach | --- | low | IA | --- | --- |
| GSM4057366 | 64 | F | duodenum | --- | low | I | KIT | E11: p.K550_V555delinsL |
| GSM4057367 | 36 | F | small intestine | --- | low | I | KIT | E11: p.L576del |
| GSM4057368 | 69 | F | duodenum | --- | low | I | KIT | E11: p.G565_N574delinsQTT |
| GSM4057369 | 60 | M | colon (rectum) | --- | low | I | KIT | E11: p.K558Q, p.L576P |
| GSM4057370 | 69 | M | duodenum | --- | low | I | KIT | E11: p.L576P |
| GSM4057371 | 45 | M | small intestine | --- | low | I | KIT | E13: p.K642E |
| GSM4057372 | 65 | F | stomach | --- | very low | IA | KIT | E11: p.D579del |
| GSM4057373 | 64 | M | stomach | --- | very low | IA | KIT | E11: p.W557_K558del |
| GSM4057374 | 60 | M | stomach | --- | very low | IA | KIT | E11: p.D579del |
| GSM4057375 | 69 | M | stomach | --- | very low | IA | KIT | E11: p.Q575_L576insPQ |
| GSM4057376 | 63 | F | stomach | --- | very low | IA | KIT | E11: p.D572_P573dup |
| GSM4057377 | 72 | F | stomach | --- | very low | IA | KIT | E11: p.Y578_D579insATQLPY |
| GSM4057378 | 78 | F | stomach | --- | very low | IA | KIT | E11: p.V559A |
| GSM4057379 | 62 | F | stomach | --- | very low | IA | KIT | E11: p.V559D |
| GSM4057380 | 69 | M | stomach | --- | very low | IA | KIT | E11: p.V559D |
| GSM4057381 | 74 | M | stomach | --- | very low | IA | KIT | E9: p.A502_Y503dup |
| GSM4057382 | 36 | M | duodenum | --- | very low | I | KIT | E11: p.V559A |

Raw data provided by Lagarde *et al.*(PMID:22167411)

| GIST patient | CINSARC | AURKA | AURKA stratification | AFIP risk | Location | site of primary tumor | local recurrence | metastasis | mutated gene | mutation |
| --- | --- | --- | --- | --- | --- | --- | --- | --- | --- | --- |
| GIST10 | C1 | 8.56 | A1 | low | 2 | small intestine | No | No | K11 | p.V560D |
| GSIT13 | C1 | 8.05 | A1 | inter | 1 | stomach | No | No | K11 | p.W557R |
| GIST15 | C1 | 7.89 | A1 | low | 1 | stomach | No | No | K11 | p.V559D |
| GIST21 | C1 | 8.66 | A1 | inter | 1 | stomach | No | No | K11 | p.L576P |
| GIST23 | C1 | 8.39 | A1 | low | 2 | small intestine | No | No | P12 | p.Y555C |
| GIST24 | C1 | 8.23 | A1 | low | 4 | peritoneum | No | No | K11 | p.T574_R586insK |
| GIST27 | C1 | 7.75 | A1 | high | 1 | stomach | No | No | K11 | p.K581_S590dup |
| GIST30 | C1 | 7.62 | A1 | inter | 1 | stomach | No | No | K11 | p.L576_R588dup |
| GIST32 | C1 | 8.09 | A1 | inter | 1 | stomach | No | No | K11 | Gp.W557R |
| GIST33 | C1 | 8.55 | A1 | very low | 1 | stomach | No | No | P18 | p.D842V |
| GIST36 | C1 | 7.61 | A1 | very low | 1 | stomach | No | No | K11 | p.V559D |
| GIST40 | C1 | 7.8 | A1 | low | 1 | stomach | No | No | K11 | p.P573_T574dup; T574dup;Q575_R586dup |
| GIST43 | C1 | 8.01 | A1 | very low | 1 | stomach | No | No | K11 | p.T574_L589dup |
| GIST44 | C1 | 8.41 | A1 | low | 1 | stomach | No | No | K11 | p.Q556_V559del |
| GIST46 | C1 | 8.6 | A1 | very low | 2 | small intestine | No | No | K11 | p.Q556_V559del |
| GIST48 | C1 | 8.14 | A1 | low | 2 | small intestine | No | No | K11 | p.M552_E561del |
| GIST49 | C1 | 8.93 | A1 | very low | 1 | stomach | No | No | K11 | p.E554_K558del |
| GSIT51 | C1 | 8.33 | A1 | very low | 1 | stomach | No | No | K11 | p.W557R |
| GIST55 | C1 | 7.72 | A1 | very low | 1 | stomach | No | No | K11 | p.D572_D579dupinsL |
| GIST60 | C1 | 8.77 | A1 | very low | 1 | stomach | No | No | P18 | p.D842V |
| GIST62 | C1 | 8.3 | A1 | very low | 1 | stomach | No | No | K11 | p.N566_P573del |
| GIST8 | C1 | 7.71 | A1 | low | 1 | stomach | No | No | K11 | p.W557_K558del |
| GIST29 | C1 | 8.48 | A1 | inter | 1 | stomach | No | No | K11 | p.D572_T574dup |
| GIST31 | C1 | 8.51 | A1 | low | 1 | stomach | No | No | P18 | P18 p.I843_D846del |
| GIST41 | C1 | 8.97 | A1 | low | 1 | stomach | No | No | P12 | P12 p.D561V |
| GIST50 | C1 | 8.36 | A1 | high | 2 | small intestine | No | No | K11 | p.M552_E554delinsK |
| GIST66 | C1 | 8.82 | A1 | low | 2 | duodenum | No | No | K11 | p.V559G |
| GIST1 | C1 | 8.12 | A1 | high | 1 | stomach | No | No | P18 | p.D842V |
| GIST54 | C1 | 9.11 | A1 | very low | 1 | stomach | No | No | P18 | p.D842V |
| GIST59 | C1 | 7.31 | A1 | very low | 1 | stomach | No | No | K11 | p.N567_L576delinsKE homo |
| GIST67 | C1 | 7.35 | A1 | low | 1 | stomach | No | No | K11 | p.V560D |
| GIST65 | C1 | 8.69 | A1 | inter | 2 | small intestine | No | No | K13 | p.K642E |
| GIST52 | C2 | 8.32 | A1 | very low | 1 | stomach | No | No | K11 | p.P573_H580ins |
| GIST18 | C2 | 9.05 | A1 | inter | 2 | duodenum | No | No | K11 | p.L576P |
| GIST64 | C2 | 8.6 | A1 | low | 2 | small intestine | No | No | K11 | p.V560D |
| GIST12 | C2 | 8.66 | A1 | high | 4 | retroperitoneum | No | No | WT | WT |
| GIST4 | C2 | 9.06 | A1 | low | 1 | stomach | No | No | K11 | p.V559D |
| GIST45 | C2 | 8.84 | A1 | very low | 1 | stomach | No | No | P18 | p.D842V |
| GIST35 | C2 | 8.85 | A1 | inter | 1 | stomach | No | No | P14 | p.N659K |
| GIST20 | C2 | 9.02 | A1 | high | 4 | abdominal wall | No | No | K11 | p.W557R |
| GIST39 | C2 | 8.88 | A1 | inter | 1 | stomach | No | Yes | K11 | p.W557_V559delins F |
| GIST22 | C2 | 9.71 | A2 | inter | 1 | stomach | No | No | P18 | p.D842V |
| GIST42 | C2 | 9.5 | A2 | low | 1 | stomach | No | No | WT | WT |
| GIST53 | C2 | 10.1 | A2 | inter | 1 | stomach | No | No | K11 | p.Q556_I563del |
| GIST5 | C2 | 9.92 | A2 | high | 1 | stomach | No | Yes | K11 | p.W557_K558 del |
| GIST63 | C2 | 10.7 | A2 | high | 3 | rectum | No | Yes | K11 | p.V560D |
| GIST11 | C2 | 9.73 | A2 | low | 2 | duodenum | No | No | K11 | p.V560A |
| GIST6 | C2 | 12.11 | A2 | high | 2 | small intestine | Yes | No | K11 | p.E554_K558del |
| GIST14 | C2 | 11.95 | A2 | inter | 4 | mesenterium | Yes | Yes | K17 | p.N822K |
| GIST16 | C2 | 9.7 | A2 | high | 2 | jejunum | No | Yes | K9 | p.A502_Y503dup |
| GIST19 | C2 | 12.01 | A2 | inter | 3 | colon | Yes | Yes | K9 | p.A502_Y503dup |
| GIST2 | C2 | 10.22 | A2 | high | 2 | small intestine | No | Yes | K11 | p.Y553_Q556del |
| GIST38 | C2 | 10.8 | A2 | high | 1 | stomach | No | Yes | K11 | p.W557_V560delinsF |
| GIST9 | C2 | 11.67 | A2 | high | 1 | stomach | Yes | Yes | K11 | p.V560D |
| GIST61 | C2 | 12.89 | A2 | high | 1 | stomach | No | Yes | P18 | p.D842V |
| GIST56 | C2 | 13.11 | A2 | high | 2 | small intestine | Yes | Yes | WT | WT |
| GIST37 | C2 | 11.2 | A2 | inter | 1 | stomach | Yes | Yes | K11 | p.W557_K558del |
| GIST28 | C2 | 10.76 | A2 | high | 1 | stomach | No | Yes | K11 | p.W557_V559delinsF |
| GIST47 | C2 | 9.64 | A2 | high | 1 | stomach | No | Yes | K11 | p.E554_D572delinsF |
| GIST58 | C2 | 10.19 | A2 | high | 1 | stomach | No | Yes | K11 | p.W557_K558delinsFP |
| GIST57 | ND | ND | ND | high | 2 | small intestine | No | Yes | K11 | p.V559D |
| GIST17 | ND | ND | ND | ND | 2 | duodenum | Yes | Yes | K11 | p.V569_L576del |
| GIST3 | ND | ND | ND | high | 1 | stomach | No | Yes | K11 | p.V560D |
| GIST26 | ND | ND | ND | inter | 4 | mediastinum | No | No | K11 | p.K558_V559delinsN homo |
| GIST34 | ND | ND | ND | very low | 2 | small intestine | No | No | K11 | p.V560D |
| GIST25 | ND | ND | ND | very low | 1 | stomach | No | No | P18 | p.D842V |
| GIST7 | ND | ND | ND | inter | 1 | stomach | No | No | K11 | p.W557_E561del |
